# GUCY2C Opposes Systemic Genotoxic Tumorigenesis by Regulating AKT-Dependent Intestinal Barrier Integrity

**DOI:** 10.1371/journal.pone.0031686

**Published:** 2012-02-22

**Authors:** Jieru Egeria Lin, Adam Eugene Snook, Peng Li, Brian Arthur Stoecker, Gilbert Won Kim, Michael Sullivan Magee, Alex Vladimir Mejia Garcia, Michael Anthony Valentino, Terry Hyslop, Stephanie Schulz, Scott Arthur Waldman

**Affiliations:** 1 Department of Pharmacology and Experimental Therapeutics, Thomas Jefferson University, Philadelphia, Pennsylvania, United States of America; 2 Divison of Biostatistics, Thomas Jefferson University, Philadelphia, Pennsylvania, United States of America; Roswell Park Cancer Institute, United States of America

## Abstract

The barrier separating mucosal and systemic compartments comprises epithelial cells, annealed by tight junctions, limiting permeability. GUCY2C recently emerged as an intestinal tumor suppressor coordinating AKT1-dependent crypt-villus homeostasis. Here, the contribution of GUCY2C to barrier integrity opposing colitis and systemic tumorigenesis is defined. Mice deficient in GUCY2C (*Gucy2c^−/−^*) exhibited barrier hyperpermeability associated with reduced junctional proteins. Conversely, activation of GUCY2C in mice reduced barrier permeability associated with increased junctional proteins. Further, silencing GUCY2C exacerbated, while activation reduced, chemical barrier disruption and colitis. Moreover, eliminating GUCY2C amplified, while activation reduced, systemic oxidative DNA damage. This genotoxicity was associated with increased spontaneous and carcinogen-induced systemic tumorigenesis in *Gucy2c^−/−^* mice. GUCY2C regulated barrier integrity by repressing AKT1, associated with increased junction proteins occludin and claudin 4 in mice and Caco2 cells in vitro. Thus, GUCY2C defends the intestinal barrier, opposing colitis and systemic genotoxicity and tumorigenesis. The therapeutic potential of this observation is underscored by the emerging clinical development of oral GUCY2C ligands, which can be used for chemoprophylaxis in inflammatory bowel disease and cancer.

## Introduction

The intestinal mucosa, from duodenum to rectum, represents the largest physiological interface separating the environment from the systemic compartment [1]–[3]. Indeed, the mucosal surface area represents the most significant anatomic portal for systemic exposure to environmental toxins and microorganisms [4]. These exposures are prevented by the epithelial barrier, in part, comprising a network of junctional complexes that anneal membranes of neighboring enterocytes and thereby restrict paracellular macromolecular exchange between mucosal and systemic compartments [3]. While the molecular components of tight junctions have been enumerated, signaling mechanisms regulating dynamic programming of their steady-state concentrations, assembly, and deployment remain incompletely defined [1]–[3], [5]. In that context, signaling by v akt murine thymoma viral oncogene homolog (AKT) plays a central role in regulating barrier dynamics [6]–[9]. Importantly, there is an emerging recognition of the contribution of barrier dysfunction to local and systemic diseases, including inflammatory bowel disease, diabetes, multiple sclerosis, food allergy, asthma, systemic genotoxicity and malignancies [1]–[3], [10], [11].

Guanylyl cyclase C (GUCY2C) is the intestinal receptor for the endogenous paracrine hormones guanylin (GUCA2A) and uroguanylin (GUCA2B) and the heat-stable enterotoxins (STs) produced by diarrheagenic bacteria [12]. Upon ligand engagement, GUCY2C converts cytosolic GTP to the second messenger cyclic GMP (cGMP), activating effector pathways principally through cyclic GMP-dependent protein kinase (PKG) [12]. GUCY2C signaling regulates intestinal fluid and electrolyte balance through PKG-dependent activation of the cystic fibrosis transmembrane conductance regulator (CFTR) [12]. Beyond fluid and electrolyte balance, GUCY2C plays a central role in crypt-villus dynamics, regulating enterocyte proliferation, differentiation, DNA damage sensing and repair, and metabolism [12]–[16]. GUCA2A and GUCA2B are the most commonly lost gene products in intestinal tumorigenesis, silencing GUCY2C signaling early in transformation [17]–[19]. Moreover, eliminating GUCY2C expression in mice (*Gucy2c^−/−^*) potentiates genetic and carcinogen-induced tumorigenesis [14], [15]. These observations suggest that GUCY2C regulates homeostatic programs along the crypt-surface axis and silencing of this paracrine hormone system through ligand loss contributes to initiation and progression of intestinal neoplasia [12]–[16].

Coordinated regulation of homeostatic processes maintaining the intestinal epithelium by GUCY2C is mediated by a molecular mechanism in which cGMP suppresses AKT phosphorylation and activation [15]. In turn, these homeostatic processes regulating epithelial integrity are essential components of molecular programs supporting mucosal barrier function [1]–[3], [10]. In the context of the established role of AKT in dynamic modulation of junctional complexes [6]–[9], we examined the impact of GUCY2C signaling on intestinal barrier integrity through AKT-dependent regulation of junctional complex components.

## Materials and Methods

A detailed description of materials and methods used in this paper can be found in *Materials and Methods S1*.

### Animal models

*Rosa-STOP^flox^*-*Guca2a-vil-Cre-ER^T2^* mice and corresponding genotype controls lacking *Rosa-STOP^flox^*-*Guca2a* insert were generated by crossbreeding *vil-Cre-ER^T2^* (provided by Robine S., Institut Curie-CNRS, France) with *Rosa-STOP^flox^*-*Guca2a* transgenic mice (Thomas Jefferson University) (see *Supplementary Information*). C57BL6 mice used in oral ST supplementation studies were purchased from NIH (NCI-Frederick). *Gucy2c^−/−^*, *Akt1^−/−^*, *Akt2^−/−^* and corresponding wild type littermate mice were bred, maintained, genotyped, and functionally characterized as described in accordance with the Thomas Jefferson University Animal Care and Use guidelines. This study was approved by The Institutional Animal Care and Use Committee of Thomas Jefferson University under approved animal protocol 315O, and 315K [15].

### DSS colitis

8 weeks old male or female mice were treated with 3 or 3.5% (w/v) DSS (Sigma-Aldrich) in their drinking water for 7 d followed by normal access to water (see *Materials and Methods S1*). Colitis severity was evaluated by body weight, mortality, and colon length, as well as histology scores. Colon length is a morphometric measure of the degree of colonic inflammation and correlates with pathologic changes related to colitis severity [20].

### Histopathology scores

Colon tissues were fixed in 10% buffered formalin, processed and embedded in paraffin. Five µm tissue sections were stained with hematoxylin-eosin (H&E). Seven sections in each mouse were evaluated by two independent investigators blinded to genotype or experimental condition. Each section was scored using the sum of a 0–4 scale in both epithelial and mesenchymal compartments based on established semiquantitative criteria (see *Table S1*) [21].

### Barrier integrity

Littermate mice were gavaged with 200 µL of FITC-dextran (75 mg/mL; 4 kD; Sigma-Aldrich) after an overnight fast. Hemolysis-free serum was analyzed for concentration of FITC-dextran by fluorimetry 90 min after gavage. Permeability of 4 kD FITC-dextran across Caco2 (ATCC) monolayers was assessed using the 24-mutiwell insert system (BD Biosciences).

### DNA oxidation

DNA was extracted from leukocytes in *Gucy2c^+/+^* and *Gucy2c^−/−^* mice, and from hepatocytes in mice treated with ST or control peptide followed by DSS administration, and stored at −20°C. DNA oxidation was estimated by quantifying 8-hydroxydeoxyguanosine employing the OxiSelect™ Oxidative DNA Damage ELISA Kit (Cell Biolabs, Inc., San Diego, CA).

### Cell culture, adenovirus and retrovirus infection

Adenovirus-expressing AKT1 and myr-AKT were provided by T. Chan (Thomas Jefferson University, PA). Retroviruses expressing shRNA against AKT1 and empty vector were produced from commercially available plasmids (Open Biosystems). After retroviral infection, Caco2 cells were selected by 8.5 µg/mL puromycin (Invitrogen).

### Immunoblot analyses

Protein was extracted from cells and tissues in M-PER reagent (Pierce) supplemented with protease and phosphatase inhibitors (Roche) and quantified by immunoblot analysis employing antibodies to: occludin, claudin 1, claudin 2, claudin 3, claudin 4, JAM-A (Invitrogen); GAPDH, phospho-AKT (Cell Signaling).

### Immunofluorescence

Distal colons were collected processed and embedded in paraffin blocks and then cut into 5 µM slides as previous described [15]. Tight junctions were stained by anti-claudin 4 antibody (Invitrogen) and epithelial cells were outlined by anti-β-catenin antibody (Santa Cruz Biotechnology). Nuclei were counterstained by DAPI.

### Microarray analyses

Microarray analyses, using the Affymetrix Mouse 430 2.0 3′-IVT platform, were performed on RNA extracted from intestine of Gucy2c^+/+^ and Gucy2c^−/−^ littermates (n = 4 each) [15]. All data is MIAME compliant and that the raw data has been deposited in GEO, GeneExpressionOmnibus: a public functional genomics data repository supporting MIAME compliant submission, http://www.ncbi.nlm.nih.gov/geo/.

### Statistical analyses

Statistical significance was determined by unpaired two-tailed Student's *t* test. Body weight and survival were analyzed by ANOVA unless otherwise specified. Results represent means ± SEM from at least 3 animals or 3 experiments performed in triplicate unless otherwise indicated.

## Results

**Figure 1:**
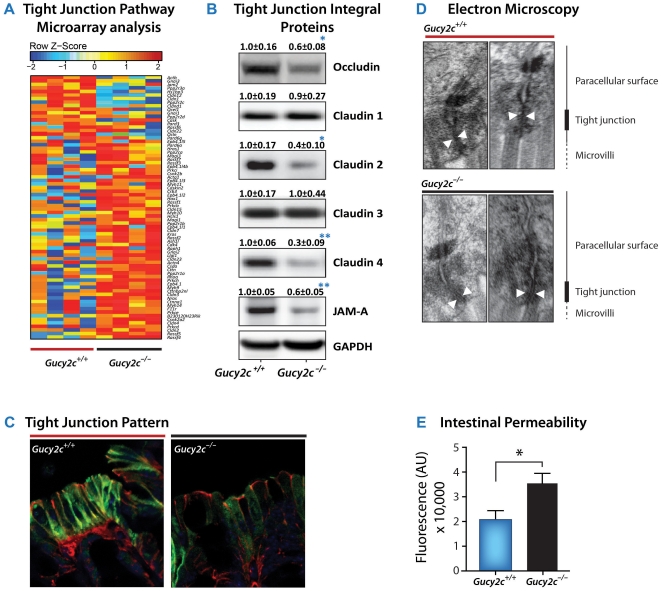
GUCY2C regulates intestinal barrier integrity. (**A**) Comparison of mRNA expression of genes associated with the canonical tight junction pathway (KEGG 04530) of *Gucy2c^+/+^* and *Gucy2c^−/−^* mice (n = 4, p = 0.029). (**B**) Immunoblot analysis of tight junction proteins from the distal colon of 12 week old *Gucy2c^+/+^* and *Gucy2c^−/−^* mice. Data represent means of n≥7±SEM. (**C**) Immunofluorescence staining of claudin 4 from the distal colon of 12 week old *Gucy2c^+/+^* and *Gucy2c^−/−^* mice. (Nucleus-DAPI, β-catenin-red, Claudin 4-green; 1,000×). (**D**) Transmission electron microscopy of tight junction complexes from jejuna of 12 week old *Gucy2c^+/+^* and *Gucy2c^−/−^* mice (25,000×). (**E**) FITC-dextran was administered by oral gavage to age-matched *Gucy2c^+/+^* and *Gucy2c^−/−^* mice after an overnight fast. Serum fluorescence was analyzed 90 min after gavage. One representative experiment of three (n = 3). *, p<0.05, **, p<0.01.

Eliminating Gucy2c signaling globally altered the expression of the family of 74 canonical tight junction-associated genes in *Gucy2c^−/−^*, compared to *Gucy2c^+/+^*, mice (Figure 1*A*; P = 0.029). Global changes in mRNA expression were associated with decreased steady-state levels of key tight junction proteins in *Gucy2c^−/−^* mice, including occludin, claudin 2, claudin 4 and JAM-A (Fig. 1*B* *, Data S1*). Reductions in these structural components were associated with reduced formation of junctional complexes between epithelial cells (Fig. 1*C*). Moroever, junctional complexes between adjacent enterocytes in *Gucy2c^−/−^* mice appeared centrally patent, compared to occluded junctions in *Gucy2c^+/+^* mice (Fig. 1*D*). These changes in junctional complexes were associated with macromolecular hyperpermeability in *Gucy2c^−/−^*, compared to Gucy2c^+/+^, mice (Fig. 1*E*).

**Figure 2:**
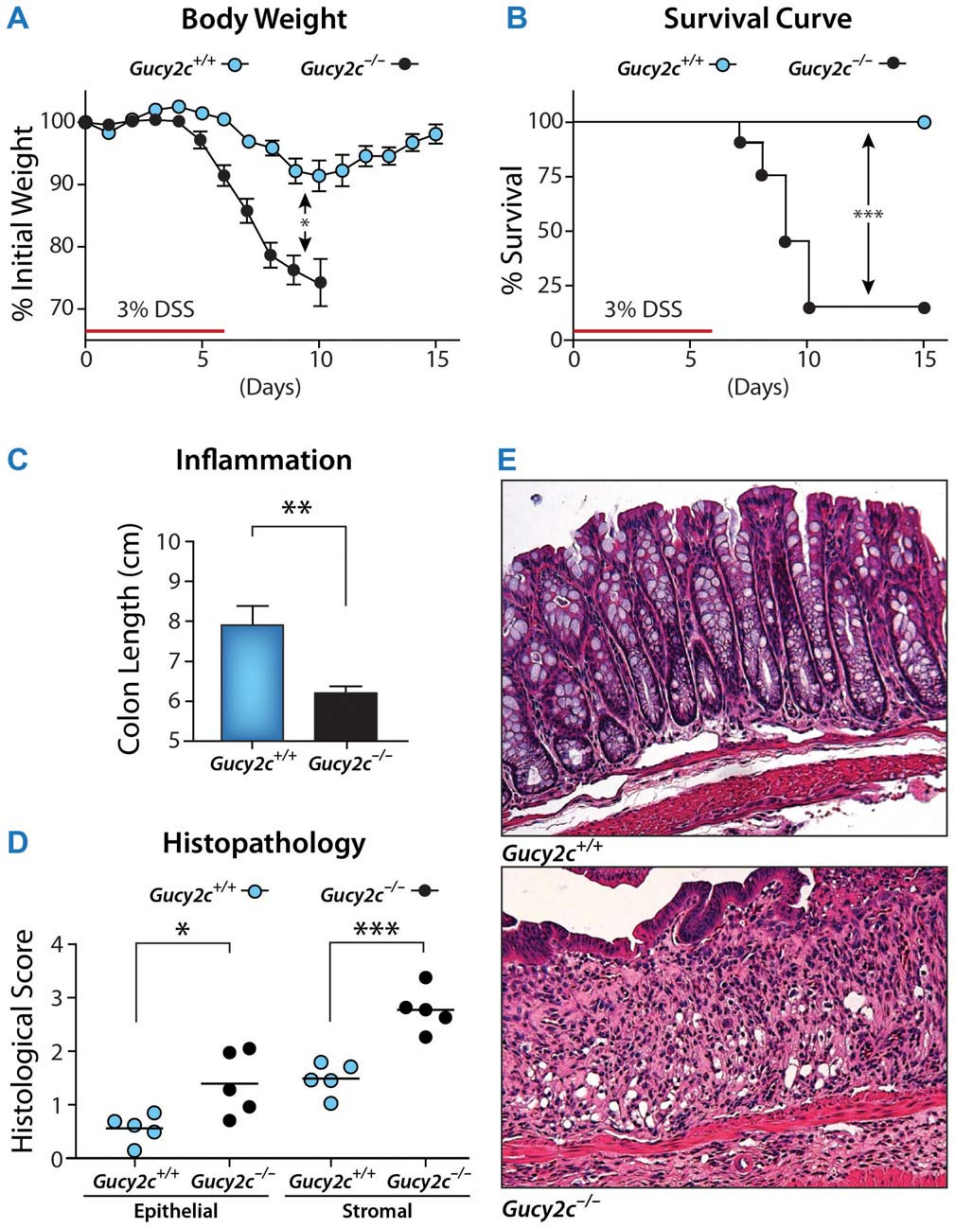
GUCY2C-deficiency increases susceptibility to DSS-induced colitis. *Gucy2c^+/+^* and *Gucy2c^−/−^* male mice were administered 3% DSS in drinking water for 7 d to disrupt the epithelial barrier and induce colitis. Severity of colitis was analyzed by (**A**) body weight (n = 11) and (**B**) survival (n≥11). (**C**) Colon length was measured from the colocecal junction to the anal verge on day 12 (5 d post-DSS exposure) to quantify chronic inflammation. (**D**) Histological sections obtained from jejunum and distal colon on day 12 were stained with H&E and scored for epithelial and mesenchymal inflammation. Each point represents one mouse. (**E**) Representative H&E colon sections (20×). Data are mean ± SD (in C) and SEM (in D) obtained from one of three independent experiments. **, p<0.01, ***, p<0.001.

Beyond its effect on physiological barrier integrity, eliminating GUCY2C signaling increased vulnerability to chemically-induced disruption of the intestinal epithelial barrier. Oral administration of dextran sulfate sodium (DSS) to mice disrupts the epithelial barrier, in part by altering expression of tight junction proteins [22], producing intestinal hyperpermeability and translocation of lumenal contents across the mucosa [1]–[3]. In turn, barrier disruption bridging the normally segregated mucosal and systemic compartments produces secondary activation of innate and adaptive immune responses, immune cell recruitment, and colitis [1]–[3]. DSS induced colitis with a 3-fold greater severity, quantified by maximum weight loss, in *Gucy2c^−/−^*, compared to *Gucy2c^+/+^*, mice (Fig. 2*A*). Also, DSS colitis was associated with >80% mortality in *Gucy2c^−/−^* mice, compared to *Gucy2c^+/+^* mice which exhibited 100% survival (Fig. 2*B*). Gross and histological pathology revealed that DSS-induced weight loss and mortality was associated with inflammatory shortening of the colon (Fig. 2*C*) and immune cell infiltration disrupting intestinal architecture (Fig. 2*D, E*) in *Gucy2c^−/−^*, compared to *Gucy2c^+/+^*, mice.

**Figure 3:**
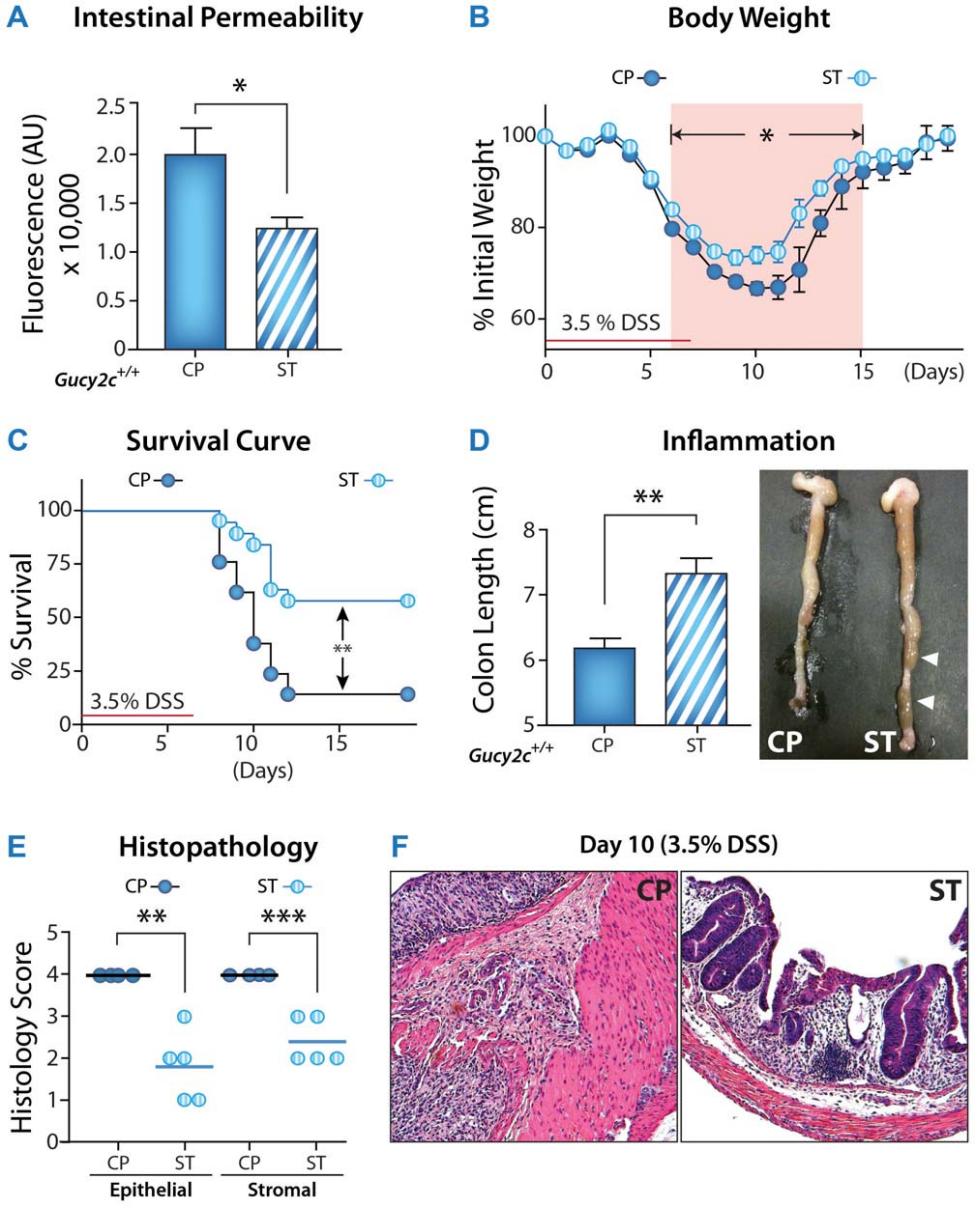
Constitutive GUCA2A expression in intestine suppresses DSS-induced colitis. Constitutive expression of GUCA2A was initiated by Cre recombinase expressed in intestinal epithelial cells after 5 days of IP tamoxifen administration in *ROSA-Guca2a*×*Vil-Cre/ER^T2^* male mice. Two days later, 3% DSS was administrated for 7 d to induce colitis. (**A**) Intestinal macromolecular permeability was assessed on day 10 (3 d post-DSS exposure) by serum fluorescence 90 min after FITC-dextran gavage (n≥5). Severity of colitis was quantified by (**B**) body weight and (**C**) survival (n≥15). Data represent one of three independent experiments. Inflammation was quantified by (**D**) colon length and (**E**) histological score on day 10 (n≥5). Each point represents one mouse. (**F**) Representative H&E colon sections (20×). Data are mean ± SEM. *, p<0.05.

**Figure 4:**
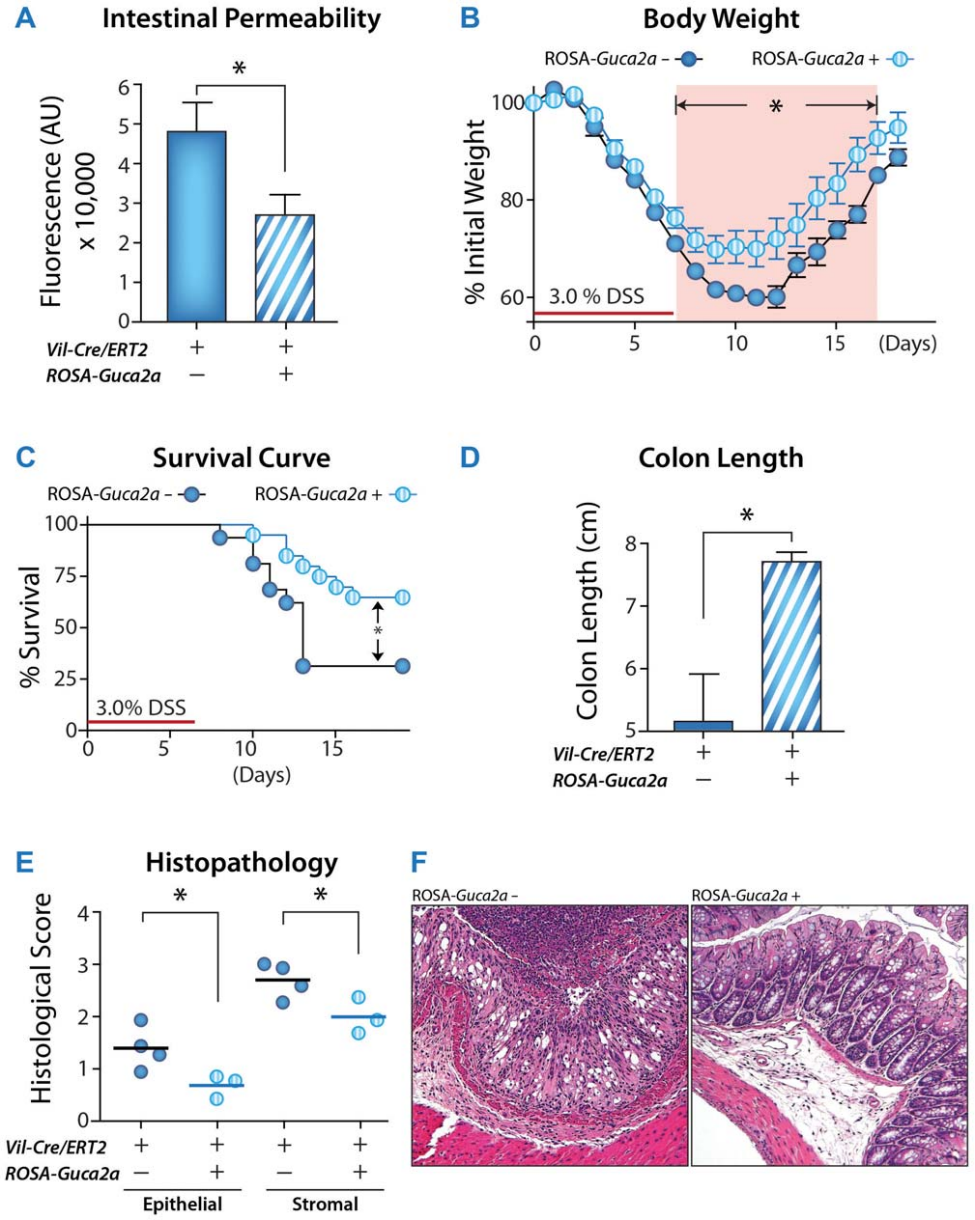
GUCY2C ligand supplementation decreases intestinal permeability and susceptibility to DSS-induced colitis. C57B6 female mice were administered control peptide (CP) or ST to examine the effect of GUCY2C activation on intestinal permeability after 14 d of oral ligand supplementation. (**A**) Serum fluorescence was analyzed 90 min after FITC-dextran gavage (n = 21) following 6 d of ligand supplementation. (**B, C**) Following 14 d of ST pre-conditioning, 3.5% DSS was administrated for 7 d to induce colitis which was quantified by (**B**) body weight and (**C**) survival. Data represent one of two independent experiments. Severity of colitis was quantified by (**D**) colon length, measured on day 10 (3 d post-DSS exposure), and gross anatomic analysis of colons from ST-, compared to CP-, preconditioned mice demonstrated normal stool formation (n = 21) and (**E**) histological score on day 10. Each point represents one mouse. (**F**) Representative H&E colon sections (20×). *, p<0.05, **, p<0.01.

Conversely, enhancing GUCY2C signaling defends the integrity of the intestinal epithelial barrier from disruption by DSS, preventing the associated colitis. *Guca2a^+^* mice constitutively express GUCA2A specifically in epithelial cells across the rostral-caudal axis of the intestine when induced by tamoxifen (see Methods). Basal mucosal integrity, quantified by intestinal permeability, was enhanced in *Guca2a^+^*, compared to control, mice (Fig. 3*A*). Further, *Guca2a^+^* mice resisted barrier disruption and colitis induced by DSS compared to wild-type mice, quantified by weight loss (Fig. 3*B*), survival (Fig. 3*C*), colon length (Fig. 3*D*), and inflammatory cell infiltration disrupting intestinal architecture (Fig. 3*E, F*). Results with the genetic model were confirmed with a complementary pharmacological model in which mice were orally administered the GUCY2C ligand ST. Like constitutive expression of the native paracrine hormone GUCA2A, oral administration of the bacterial peptide ST enhanced basal barrier integrity (Fig. 4*A*) and opposed barrier disruption and colitis induced by DSS (Fig. 4*B–F*).

**Figure 5:**
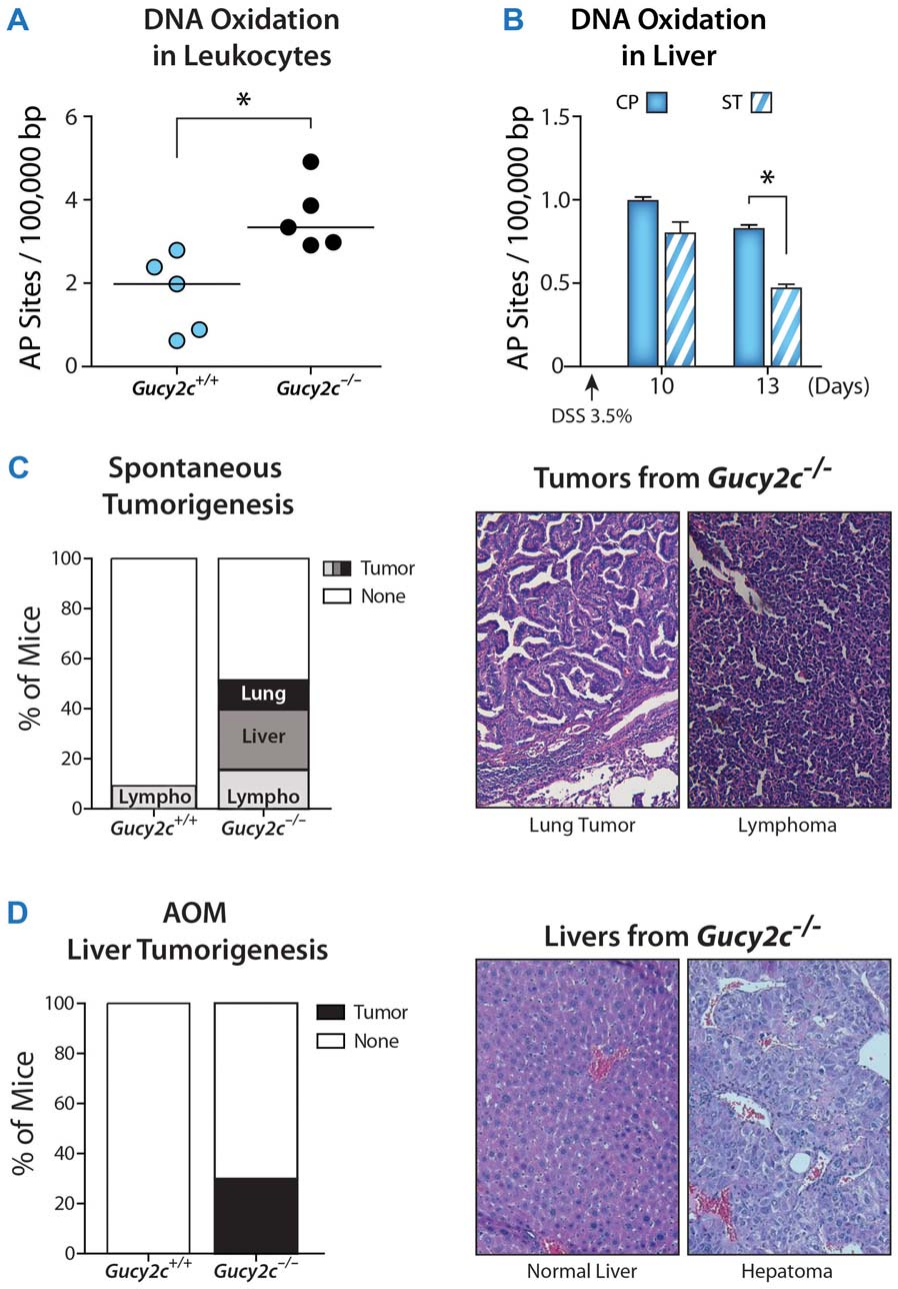
GUCY2C modulates systemic genotoxicity and tumorigenesis. (**A**) Eliminating GUCY2C increases basal DNA oxidation in leukocytes. Each point represents one mouse. (**B**) Oral ST supplementation decreases DSS-induced hepatic genotoxicity. *Gucy2c^+/+^* mice were preconditioned with oral ST for 6 d, and then treated with 3.5% DSS for 7 d, followed by quantification of DNA oxidation in liver on days 10 (n≥5) and 13 (n≥2; 3 and 6 d post-DSS exposure). Data represent mean ± SD. (**C**) *Gucy2c^−/−^* mice exhibited a higher incidence of spontaneous tumors comparing with age-matched 2-year-old *Gucy2c^+/+^* mice (n≥14, p = 0.02). (**D**) The carcinogen AOM induced hepatoma in *Gucy2c^−/−^*, but not *Gucy2c^+/+^*, mice (n≥10, p = 0.04). C and D are analyzed by two-sided Fisher's exact test. *, p<0.05.

There is an established relationship between disruption of the epithelial barrier associated with local inflammation and the production of systemic genotoxic insult, including DNA damage reflecting reactive oxygen species that underlie tumor initiation and progression [11]. Impairment of basal epithelial barrier integrity in *Gucy2c^−/−^* mice was associated with increased extra-intestinal oxidative DNA damage in leukocytes, compared to *Gucy2c^+/+^* mice (Fig. 5*A*). Conversely, enhancing barrier integrity with oral ST reduced hepatic oxidative DNA damage in mice treated with DSS (Fig. 5*B*). Impaired basal epithelial barrier integrity producing systemic genotoxicity was associated with spontaneous extra-intestinal tumorigenesis, including tumors in mesenteric lymph nodes, livers, and lungs, in 50% of *Gucy2c^−/−^* mice, but in only 10% of *Gucy2c^+/+^* mice (Fig. 5*C*). Moreover, elevated basal systemic genotoxicity reflecting barrier corruption potentiated induction of hepatoma by the chemical carcinogen azoxymethane (AOM) in *Gucy2c^−/−^*, but not in *Gucy2c^+/+^*, mice (Fig. 5*D*).

**Figure 6:**
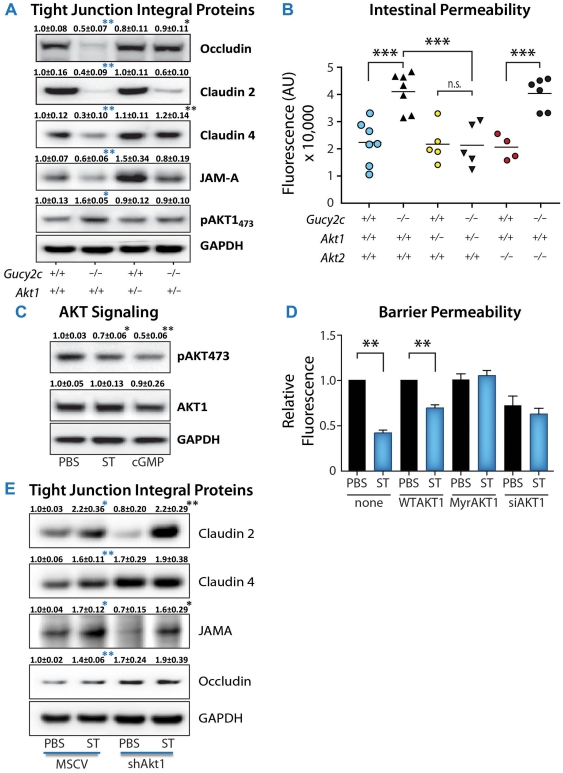
GUCY2C regulation of barrier integrity is AKT1-dependent. (**A**) Immunoblot analysis of tight junction proteins from jejuna of 12 week-old mice (n≥7). Data represent means ± SEM. Blue asterisks compare results to *Gucy2c^+/+^*, black asterisks compare results to *Gucy2c^−/^*. (**B**) Intestinal permeability was examined by serum fluorescence 90 min after FITC-dextran gavage. Each point represents one mouse. (**C**) Regulation of AKT phosphorylation by GUCY2C signaling in Caco2 human colon cancer cells was examined by immunoblot analysis. Data represent means ± SEM of 3 experiments done in duplicate. (**D**) AKT1 signaling in Caco2 cells was manipulated by adenovirus-delivered AKT1 (WTAKT1), constitutive active AKT1 mutant (MyrAKT1), or siRNA against AKT1 (siAKT1). Two days after infection, cells were treated with ST for 6 d. Barrier permeability was examined by FITC-dextran diffusion. Data represent mean ± SEM obtained from one of five experiments done in triplicate. (**E**) Tight junction protein expression was examined by immunoblot analysis in Caco2 cells stably expressing empty vector (MSCV) or shRNA against AKT1 (shAKT1) after 6 d of ST treatment. Data represent means ± SEM of three experiments done in duplicate. Blue asterisks compare results to MSCV-PBS, black asterisks compare results to MSCV-ST. *, p<0.05, **, p<0.01, ***, p<0.001. In C–E, statistical analyses were compared to PBS control.

GUCY2C modulates homeostatic processes by opposing AKT1 phosphorylation and downstream signaling in intestine and colorectal cancer [15]. In the context of established AKT modulation of junctional complexes [6]–[9], steady-state levels of the tight junction proteins occludin and claudin 4, but not claudin 2 or JAM-A, were restored to those in wild type epithelial cells by normalizing phospho-AKT1 levels in *Gucy2c^−/−^Akt1^+/−^* mice (Fig. 6*A*). Restoration of occludin and claudin 4 was associated with reconstitution of intestinal barrier integrity characteristic of wild type mice by reducing AKT1, but not AKT2, expression in *Gucy2c^−/−^* mice (Fig. 6*B*).

Normalizing phospho-AKT1 levels in *Gucy2c^−/−^Akt1^+/−^* mice mimics the effect of GUCY2C on AKT1 in Caco2 human intestinal epithelial cells, an established *in vitro* model of the intestinal barrier [23]. Stimulating GUCY2C with ST reduced phospho-AKT1, an effect recapitulated by the downstream GUCY2C effector, cGMP (Fig. *6C*). ST enhanced the barrier properties of these cells, decreasing the permeability of Caco2 monolayers (Fig. 6*D*), reproducing the *in vivo* effect of ST on intestinal permeability (Fig. 4*A*). The effect of ST on Caco2 permeability was mimicked by reducing AKT1 expression using a target-specific siRNA, recapitulating the effects of genetic knockdown of AKT1 in *Gucy2c^−/−^Akt1^+/−^* mice (Fig. *4B*). Conversely, the effect of ST on Caco2 permeability was eliminated by a constitutively active AKT1 mutant, MyrAKT1 (Fig. 6*D*). Moreover, reducing the expression of AKT1 in Caco2 cells using a target-specific shRNA reproduced the effects of GUCY2C signaling on steady-state levels of occludin and claudin 4, but not claudin 2 or JAM-A (Fig. 6*E*), precisely recapitulating the effects of GUCY2C and AKT1 on junctional proteins in *Gucy2c^−/−^Akt1^+/−^* mice (Fig. 6A).

## Discussion

Disruption of intestinal barrier integrity has profound pathophysiological consequences and there is an emerging recognition of the contribution of the “leaky gut” and intestinal hyperpermeability to human disease [1]–[3], [10], [24]. In part, intestinal barrier integrity reflects dynamic modulation of tight junction complexes through regulation of component proteins, including intracellular steady-state concentrations and their assembly and membrane deployment [2], [3]. These complexes are rate-limiting components in transepithelial transport and primary determinants of mucosal permeability [2], [3], [10]. Acquired or inherited impairment of this barrier is at least necessary, if not sufficient, for abnormal subepithelial exposure to lumenal antigens that provoke immune responses underlying inflammatory bowel disease [1], [2]. Beyond these local effects, epithelial barrier dysfunction leading to cross-compartmental exposure to environmental antigens may be one necessary precondition for the development of systemic multi-organ autoimmune diseases including type I diabetes, multiple sclerosis, asthma, food allergy, hypersensitivity and cancer [1]–[3], [10], [24]. These considerations underscore an unmet clinical need for novel therapeutic approaches that defend intestinal barrier integrity to prevent systemic disease.

While GUCY2C was originally identified as the intestinal receptor for the diarrheagenic bacterial STs, its role in modulating physiological fluid and electrolyte secretion emerged following the discovery of the endogenous paracrine hormones GUCA2A and GUCA2B [25]. Beyond fluid and electrolyte balance, recent studies have revealed a role for GUCY2C in regulating homeostatic programs that shape the dynamic crypt-villus axis. GUCY2C regulates key cell cycle circuits, restricting cell division as epithelial cells transition from the proliferating crypt to the differentiated villus compartment [13]–[16]. Also, GUCY2C drives differentiation along the secretory lineage, amplifying the production of goblet, Paneth and enteroendocrine cells [13]. Further, GUCY2C signaling shifts metabolic programming from glycolytic to mitochondrial oxidative metabolism as cells transition from proliferating to differentiated compartments [15]. Moreover, GUCY2C modulates DNA damage sensing and repair, protecting chromosomal integrity [13], [14]. Disruption of these homeostatic processes, reflecting universal early loss of expression of GUCA2A and GUCA2B which silences GUCY2C, contributes to hyperproliferation and chromosomal instability underlying intestinal tumorigenesis in animals and humans [16]–[19].

The present studies extend this homeostatic role of GUCY2C beyond proliferation, differentiation, migration, DNA damage sensing and repair, and metabolism to maintenance of macromolecular permeability and the intestinal barrier. Elimination of GUCY2C signaling produced basal macromolecular hyperpermeability and potentiated barrier disruption resulting in colitis induced by DSS [26]. Conversely, genetic or pharmacological induction of GUCY2C signaling reduced basal permeability and DSS-induced hyperpermeability and inflammation. Results in mouse models were recapitulated in monolayers of human intestinal epithelial cells *in vitro*, in the absence of other confounding (patho)physiological mechanisms like inflammation, demonstrating a primary mechanistic role for GUCY2C in regulating the epithelial barrier and macromolecular permeability. Moreover, protection of the epithelial barrier by GUCY2C *in vivo* and *in vitro* was associated with alterations in steady state concentrations of tight junction proteins, including occludin and claudin 4. Regulation of steady-state concentrations of these components are one established mechanism modulating the dynamic assembly and deployment of tight junction complexes, barrier integrity and macromolecular permeability [1]–[3], [10], [27].

Integration of crypt-villus homeostatic programs mediated by GUCY2C is coordinated through AKT1 [15]. Ligand activation of GUCY2C and accumulation of cGMP produces dephosphorylation of AKT1, modulating downstream signaling circuits regulating proliferation and metabolism [15]. In the present study, homeostatic regulation of epithelial barrier function and permeability by GUCY2C was mediated by AKT1. In that context, specifically reducing AKT1, but not AKT2, blocked alterations in occludin and claudin 4, but not JAM-A and claudin 2, and macromolecular hyperpermeability induced by eliminating GUCY2C signaling in mice. Further, in monolayers of human intestinal epithelial cells *in vitro*, eliminating AKT1 expression induced steady state levels of occludin and claudin 4, but not JAM-A and claudin 2, and barrier function to levels achieved by ST activation of GUCY2C. Moreover, a constitutively activated form of AKT1 produced resistance to, while eliminating AKT1 expression from these cells mimicked, the effects of GUCY2C activation on epithelial cell permeability. Together, these results demonstrate a key mechanistic role for suppression of AKT1 signaling in the regulation of epithelial cell barrier function by GUCY2C. These results reinforce the established role of AKT in regulating the expression of junctional proteins, disrupting epithelial cell membrane complexes, and inducing barrier dysfunction [6]–[9]. Further, they identify occludin and claudin 4, but not JAM-A and claudin 2, as specific downstream targets of AKT1 contributing to the regulation of epithelial barrier permeability *in vitro* and *in vivo*. They underscore the integrated homeostatic role of GUCY2C signaling, centrally coordinated through AKT, in regulating intestinal epithelial structure and function [12]–[15].

There is an established mechanistic relationship between disruption of the intestinal epithelial barrier, inflammation resulting from systemic immune exposure to normally compartmentalized lumenal antigens, and the generation of reactive oxygen species producing DNA damage and genotoxic stress [28]. In turn, reactive oxygen species producing DNA damage comprise one mechanism contributing to tumorigenesis generally, and to the evolution of colon cancer in the context of inflammatory bowel disease, specifically [11], [28]. Importantly, there is emerging recognition that this oxidative stress extends beyond the primary site of barrier disruption and inflammation, and is disseminated systemically [11]. Indeed, genetic or chemical disruption of the intestinal barrier associated with inflammation produces systemic DNA damage and genotoxic stress, including in circulating leukocytes [11]. Here, barrier disruption and hyperpermeability produced by silencing GUCY2C was associated with elevated levels of oxidative DNA damage in circulating leukocytes. Conversely, defending barrier integrity and restricting permeability by activating GUCY2C with oral ST decreased systemic genotoxicity in hepatocytes produced by DSS. Further, systemic genotoxicity produced by barrier disruption reflecting GUCY2C silencing was associated with increased spontaneous and carcinogen-induced extra-intestinal tumorigenesis, specifically in lung, liver and lymph nodes. In the context of the role of GUCY2C as a tissue-specific tumor suppressing receptor [12], the present observations expand that function beyond transformation in the colorectum [29], to neoplasia in lymph nodes, liver, and lung through maintenance of epithelial barrier integrity. Moreover, it is tempting to speculate that loss of paracrine hormone expression and silencing of GUCY2C in inflammatory bowel disease [30] produces systemic genotoxicity which contributes to the pattern of extra-intestinal cancer in these patients specifically in lymph nodes, liver, and lung [31], [32].

The present study further clarifies the evolving role of the GUCY2C paracrine hormone system in modulating the intestinal barrier. A role for GUCY2C in regulating epithelial barrier function was recently demonstrated, and the present work confirms that phenomenon [33]. Those studies revealed that silencing GUCY2C disrupted the barrier and induced inflammation, associated with production of interferon gamma and IL-12 [33]. In turn, those events were associated with increased expression of epithelial cell myosin light chain kinase, an established regulator of tight junction structure and function, and suppression of JAM-A and claudin 2 expression. These phenomena were presumed to be the molecular mechanisms mediating GUCY2C-dependent barrier disruption. However, canonical regulation of myosin light chain phosphorylation by cGMP is mediated by myosin light chain phosphatase, rather than myosin light chain kinase [34]. Moreover, these observations are complicated by the contribution of inflammatory cytokines, including interferon gamma, to regulation of expression of myosin light chain kinase in intestinal epithelial cells *in vivo* and *in vitro* [2], [3]. Thus, the contribution of these components may not reflect the primary mechanism underlying GUCY2C regulation of barrier integrity but, rather, an epiphenomenon related to the associated inflammation [33]. In contrast, the present study demonstrates a mechanistic role for GUCY2C as a primary modulator of the intestinal epithelial barrier, mediated by AKT1, occludin and claudin 4, but not JAM-A and claudin 2, in mouse models *in vivo*, and in human intestinal cell monolayers *in vitro* in the absence of confounding by inflammation.

Paradoxically, subsequent work from the same group suggested that eliminating GUCY2C signaling protects the intestinal mucosa from inflammatory injury [35]. In that study, barrier disruption by DSS induced greater inflammation, tissue damage, and mortality in *Gucy2^+/+^*, compared to *Gucy2c^−/−^*, mice [35]. These results stand in striking contradistinction to earlier reports by this group [33]. Indeed, while baseline inflammation was elevated in *Gucy2c^−/−^* mice previously [33], here these mice exhibited reduced levels of inflammatory cytokines including interferon gamma [35]. Similarly, while barrier disruption produced exaggerated responses in *Gucy2c^−/−^* mice previously [33], here barrier disruption produced disease of greater severity in *Gucy2c^+/+^* mice [35]. Beyond the inconsistencies in these observations from the same group, they stand in striking contrast to the results presented herein, demonstrating that GUCY2C signaling defends mucosal integrity against barrier disruption and colitis produced by DSS. The importance of these inconsistencies can best be appreciated by their respective therapeutic implications. On the one hand, if cGMP signaling exacerbates barrier disruption resulting in inflammation, then GUCY2C ***antagonists*** could be therapeutic in inflammatory bowel disease. Conversely, if cGMP signaling defends the barrier and prevents ensuing inflammation, then GUCY2C ***agonists*** could be a significant addition to the therapeutic armamentarium against inflammatory bowel disease.

While the source remains unclear, one possibility for these and past inconsistencies [14], [36] is the animal models used for genetic comparisons [37]. Here, all comparisons between *Gucy2c^−/−^* and *Gucy2c^+/+^* mice were performed with matched colony mates. This experimental design reflects our observation that wild type mice obtained commercially exhibit increased susceptibility to DSS compared to wild type mice from our colony (See Figure S1). If earlier studies [35], [36] compared the behavior of *Gucy2c^−/−^* or *Guca2a* ^−/−^ mice to commercially-available wild type mice, inconsistent results could reflect differences in genetic or environmental characteristics beyond the specific genes targeted in the models. The present study demonstrates the role of the GUCY2C paracrine axis in barrier regulation using multiple genetic models that either eliminate receptor circuits or constitutively express paracrine ligands, with appropriate intra-colony controls, complemented by studies of oral GUCY2C ligand efficacy conducted in wild type mice from an independent colony. This evidence strongly supports a role for the GUCY2C paracrine axis in maintaining barrier integrity, and the therapeutic value of GUCY2C ligands in preventing and treating barrier disruption and inflammatory bowel disease.

Beyond proliferation, metabolism, and chromosomal integrity, the present studies reveal an additional dimension of AKT1-dependent epithelial homeostatic regulation by GUCY2C. Indeed, it is tempting to speculate that one essential function of GUCY2C signaling is maintenance of intestinal barrier integrity. In that context, regulation of epithelial tight junctions contributing to macromolecular permeability described here can be added to other established GUCY2C functions, including fluid and electrolyte secretion accelerating lumenal clearance; differentiation of Paneth cells producing antimicrobial peptides; and differentiation of goblet cells producing intestinal mucus that contribute to the separation of systemic and environmental compartments across the mucosal interface [13]. The pathophysiological significance of this role in intestinal barrier protection is underscored by the impact of GUCY2C signaling on colitis and systemic genotoxicity and tumorigenesis. In turn, these considerations highlight the translational opportunities for GUCY2C ligands in the prevention and treatment of inflammatory bowel disease and extra-intestinal malignancies. This therapeutic potential is underscored by the recent advancement of the oral GUCY2C ligand linaclotide for FDA approval for the treatment of constipation-type irritable bowel syndrome [38].

## Supporting Information

Figure S1 **Environmental effects on DSS-induced colitis.** 8 week old C57JBL6 mice purchased from NCI (black circles) and in-house C57JBL6 mice generated from Gucy2c+/− colony (blue circles) were fed with 3% DSS for 7 d followed by regular drinking water. Body weight (n≥11) was measured as an indicator for severity of colitis. Values are given as percentage of body weight on day 0±SEM. *, p<0.05.
(PDF)

Table S1 **Histopathology score table.** Semiquantitative histopathological scoring system based on the criteria presented in the table was used by two independent investigators blinded to genotypes or experimental condition to assess severity of colitis. Scores from epithelium and mesenchyme were summed to determine the degree of inflammation for each section. Mean scores of at least seven sections per animal were presented in figures.
(PDF)

Materials and Methods S1 **Detailed experimental procedures and reagents used in this manuscript.**
(PDF)

Data S1 **Result of KEGG pathway 04530 (Tight Junction) between C57Bl6** ***Gucy2c^+/+^*** **and** ***Gucy2c^−/−^*** **mice.**
(HTML)
