# Supplementary figures and images for: GUCY2C Opposes Systemic Genotoxic Tumorigenesis by Regulating AKT-Dependent Intestinal Barrier Integrity
Source: PLoS One. 2012 Feb 22;7(2):e31686. doi: 10.1371/journal.pone.0031686 (PMC3284579; doi:10.1371/journal.pone.0031686)

Figure S1

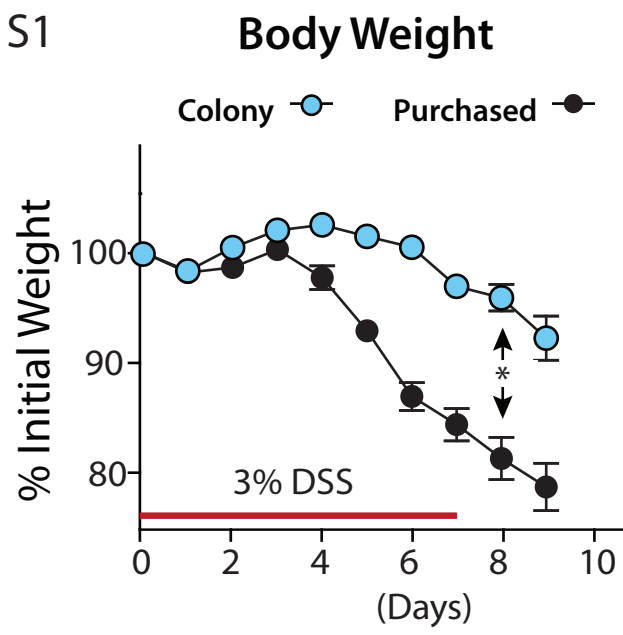

Supplement: Figure S1 — Environmental effects on DSS-induced colitis. 8 week old C57JBL6 mice purchased from NCI (black circles) and in-house C57JBL6 mice generated from Gucy2c+/− colony (blue circles) were fed with 3% DSS for 7 d followed by regular drinking water. Body weight (n≥11) was measured as an indicator for severity of colitis. Values are given as percentage of body weight on day 0±SEM. *, p<0.05. (PDF) [file pone.0031686.s001.pdf]
