# Supplementary material for: GUCY2C Opposes Systemic Genotoxic Tumorigenesis by Regulating AKT-Dependent Intestinal Barrier Integrity
Source: PLoS One. 2012 Feb 22;7(2):e31686. doi: 10.1371/journal.pone.0031686 (PMC3284579; doi:10.1371/journal.pone.0031686)
Supplement: Table S1 — Histopathology score table. Semiquantitative histopathological scoring system based on the criteria presented in the table was used by two independent investigators blinded to genotypes or experimental condition to assess severity of colitis. Scores from epithelium and mesenchyme were summed to determine the degree of inflammation for each section. Mean scores of at least seven sections per animal were presented in figures. (PDF) [file pone.0031686.s002.pdf]

Supplementary Table 1

Score	Epithelium	Score	Inflammatory Cell Infiltration
0	Normal	0	No infiltration
1	Loss of < 50% of crypts	1	Infiltration around crypt bases
2	Loss of > 50% of crypts	2	Thickening of the mucosa with edema
3	The lamina propria is covered with a single layer of epithelium	3	Infiltration of the submucosa (< 50%)
4	Erosions	4	Infiltration of the submucosa (> 50%)
