# Supplementary material for: GUCY2C Opposes Systemic Genotoxic Tumorigenesis by Regulating AKT-Dependent Intestinal Barrier Integrity
Source: PLoS One. 2012 Feb 22;7(2):e31686. doi: 10.1371/journal.pone.0031686 (PMC3284579; doi:10.1371/journal.pone.0031686)
Supplement: Materials and Methods S1 — Detailed experimental procedures and reagents used in this manuscript. (PDF) [file pone.0031686.s003.pdf]

Supplementary Information

Materials and Methods

Animal models

Gucy2c$^{-/-}$ mice were generated by neomycin resistance gene insertion as previously described.[1,2] *Gucy2c*$^{-/-}$ mice used in the experiments were backcrossed to C57BL/6 mice (The Jackson Laboratory) for 14 generations. Sibling *Gucy2c*$^{+/+}$ and *Gucy2c*$^{-/-}$ mice produced from at least three different breeding pairs of the 14th generation of *Gucy2c*$^{+/-}$ mice were used to generate colony mice used in the experiments. *Akt1*$^{+/-}$*Gucy2c*$^{+/+}$ and *Akt1*$^{+/-}$*Gucy2c*$^{-/-}$ mice were generated by crossing *Akt1*$^{-/-}$*Gucy2c*$^{+/-}$ male with *Akt1*$^{+/+}$*Gucy2c*$^{+/-}$ female mice, *Akt1*$^{-/-}$*Gucy2c*$^{+/+}$ male with *Akt1*$^{+/+}$*Gucy2c*$^{+/+}$ female mice or *Akt1*$^{-/-}$*Gucy2c*$^{-/-}$ male with *Akt1*$^{+/+}$*Gucy2c*$^{-/-}$ female mice. *Akt*$^{-/-}$*Gucy2c*$^{+/+}$ and *Akt*$^{-/-}$*Gucy2c*$^{-/-}$ mice were generated from *Akt1*$^{-/-}$ male and *Akt1*$^{+/-}$ female with specific homozygous *Gucy2c* genotype.[3] *Rosa-STOP*^{flox}*-Guca2a* mice were generated by standard transgenic procedures in the Thomas Jefferson University transgenic mouse facility.[4] Expression of the GUCY2C ligand, guanylin (GUCA2A), is regulated by the *Rosa26* promoter followed by a STOP codon flanked by two *loxP* sites upstream of full length *Guca2a* in *Rosa-STOP*^{flox}*-Guca2a* mice. Removal of the STOP codon by Cre recombinase activates constitutive transcription of *Guca2a* driven by the *Rosa26* promoter. The murine villin promoter targets stable and homogeneous expression of transgenes in small and large intestine along the crypt-villus axis, in differentiated enterocytes, as well as in the immature, undifferentiated cells of the crypt. *Villin-Cre*-ER^{T2} mice were obtained from S. Robine (Institut Curie, Paris, France).[5] *Villin-Cre*ER^{T2} mice express Cre recombinase in intestinal epithelial cells after intraperitoneal injection of tamoxifen (20 mg/Kg BW) for 5 consecutive days.[5] *Villin-*

*Cre*ER^{T2} mice were crossed with *Rosa-STOP^{flox}-Guca2a* mice to generate heterozygous *Rosa-STOP^{flox}-Guca2a-vil-Cre-ER^{T2}* mice. *Villin-Cre*ER^{T2} mice were paired with heterozygous *Rosa-STOP^{flox}-Guca2a-vil-Cre-ER^{T2}* mice to produce *Rosa-STOP^{flox}-Guca2a-vil-Cre-ER^{T2}* mice and corresponding littermate controls lacking the *Rosa-STOP^{flox}-Guca2a* transgene. Both *Villin-Cre*ER^{T2} and *Rosa-STOP^{flox}-Guca2a* mice were in the C57BL/6 background. All mice were housed in light-cycled and climate-controlled conventional animal facilities. All experiments were performed in compliance with the Thomas Jefferson University Animal Care and Use Guidelines.

Gucy2c genotype was confirmed by primers (forward: 5'-AGGTCATGACGTCACTGCTGGGCC-3'; reverse: 5'-TGTCCAGTCCTTCCTC CACAG-3'; neomycin: 5'-GGTGGGCTCTATGGCTTC-3').[1, 2] (Li, AJP 2007, Lin, Gastro 2010). *Akt1* genotype was confirmed by PCR with *Akt1* primers (Cp325: 5'-GCTCCATAAGCACACCTTCAG-3'; Cp326: 5'-GTGGATGGGAA TATGTGCGAG-3'; Cp328: 5'-ACAAGCTCTTCTTCCACCTGTC-3').[3] *Rosa-STOP^{flox}-Guca2a* genotype was confirmed by PCR with primers (forward: 5'-CCGCCGTTGTTGTTATTGTAG-3'; reverse: 5'-GTTGTGGTGATAGGTGGCAAG-3'). *Villin-Cre*-ER^{T2} genotype was confirmed by primers (forward: 5'-GAAAATGCTTCTGTCCGTTTG-3'; reverse: 5'-ATTGCTGTCACTTGGTCGTG-3').

DSS colitis

Age matched *Gucy2c*$^{+/+}$ and *Gucy2c*$^{-/-}$ mice were treated with 3.0% (w/v) DSS (Sigma-Aldrich) in the drinking water for 7 d followed by normal access to water.[6] Preliminary dose-finding experiments exploring the colitic effects of 0-5% DSS permitted selection of DSS doses for these studies that achieved inflammation within 7 d. *Rosa-STOP^{flox}-Guca2a-vil-Cre-ER^{T2}*, and corresponding littermate control *Villin-Cre*ER^{T2}, mice were injected intraperitoneally with tamoxifen (20 mg/Kg BW) for 5 d. Gene expression was analyzed 7 d after the last tamoxifen

treatment by mRNA and protein expression. DSS (3.0%; w/v) was administrated in the drinking water for 7 d followed by normal access to water. For ligand supplementation experiments, 8 wk old C57BL/6 mice were purchased from NCI and gavaged with ST or control peptide for 14 d before receiving 3.5% DSS for 7 d. Body weight and physical activity were monitored daily.

Barrier integrity

Mice were gavaged with 200 µL of FITC-dextran (75 mg/mL; 4kD; Sigma-Aldrich) and blood was collected 90 min later by cardiac puncture and added to EDTA-coated tubes. Hemolysis-free serum was collected by centrifugation and evaluated immediately for fluorescence (485/525 nM) in black opaque plates. FITC-dextran (7.5 mg/mL) was used as 90% for gain adjustment. Background fluorescence was determined by serum from mice without FITC-dextran gavage.[7]

Caco2 human colon cancer cells were infected with wtAKT1 or MyrAKT1 delivered by adenovirus, or stably transfected with shAKT1 and MSCV empty vector delivered by retrovirus, grown into monolayers on 24-well PET transwell permeable inserts (BD Biosciences, 1.0 µm pore size, coated with fibrillar collagen), and treated with ST (1 µM) and IBMX (50 µM) or IBMX alone for 6 d. Complete growth media and reagents were exchanged every other day. After 6 d of treatment, 40 µL of 75 mg/mL FITC-dextran was added into the upper chamber containing 400 µL of complete growth media, while 800 µL of complete growth media was present in the lower chamber. FITC-dextran (7.5 mg/mL) was used as 90% for gain adjustment. After 60 min of incubation, the fluorescence of the media in the bottom chamber was assessed to determine permeability.

Electron microscopy

Intestinal sections 10 cm after the pyloric sphincter were cleaned, collected and fixed with 2.5% glutaraldehyde/2% formaldehyde with 0.1 M sodium cacodylate and stored at 4°C until embedding. Post-fixation with 2% osmium tetroxide and an increasing gradient dehydration step using ethanol and propylene oxide was followed by embedding in LX-112 medium (Ladd Research). Sections (90 nm) were placed on uncoated copper grids and stained with 0.2% lead citrate and 1% uranyl acetate. Images were collected in a blinded fashion with a JEOL-1010 electron microscope employing 80 kV of acceleration.[3]

Microarray analysis

Microarrays, using the Affymetrix Mouse 430 2.0 3'-IVT platform, were run on RNA extracted from colon and small intestine of *Gucy2c*$^{+/+}$ and *Gucy2c*$^{-/-}$ littermates (n=4).[3] The quality of RNA, as well as hybridization to chips, was assessed by hybridization levels of spike-in controls, 3'-5' RNA degradation profiles, and modeling the probe-level data to investigate consistency across arrays.[8] Robust Multichip Analysis (RMA[9]) was used to background-correct and normalize the probe-level data and summarize it at the probeset level. The processed data was evaluated for differential expression of genes using permutations tests and libraries from the Bioconductor project.[10] Global tests of differential expression of groups of genes (genesets) determined from KEGG pathways or from prior biological interest were performed as described.[11] Gene ontology-based scoring was performed using pathway sets curated from the Biocarta and KEGG databases (www.biocarta.com; http://www.genome.jp/kegg/ pathway.html).[12]

Supplemental References

1. Schulz S. Targeted gene disruption in the development of mouse models to elucidate the role of receptor guanylyl cyclase signaling pathways in physiological function. Methods 1999;19:551-8.
2. Schulz S, Green CK, Yuen PS, Garbers DL. Guanylyl cyclase is a heat-stable enterotoxin receptor. Cell 1990;63:941-8.
3. Lin JE, Li P, Snook AE, Schulz S, Dasgupta A, Hyslop TM, Gibbons AV, Marszlowicz G, Pitari GM, Waldman SA. The hormone receptor GUCY2C suppresses intestinal tumor formation by inhibiting AKT signaling. Gastroenterology 2010;138:241-54.
4. Fernandes-Alnemri T, Yu JW, Juliana C, Solorzano L, Kang S, Wu J, Datta P, McCormick M, Huang L, McDermott E, Eisenlohr L, Landel CP, Alnemri ES. The AIM2 inflammasome is critical for innate immunity to Francisella tularensis. Nat Immunol 2010;11:385-93.
5. el Marjou F, Janssen KP, Chang BH, Li M, Hindie V, Chan L, Louvard D, Chambon P, Metzger D, Robine S. Tissue-specific and inducible Cre-mediated recombination in the gut epithelium. Genesis 2004;39:186-93.
6. Okayasu I, Hatakeyama S, Yamada M, Ohkusa T, Inagaki Y, Nakaya R. A novel method in the induction of reliable experimental acute and chronic ulcerative colitis in mice. Gastroenterology 1990;98:694-702.
7. Gibson DL, Montero M, Ropeleski MJ, Bergstrom KS, Ma C, Ghosh S, Merkens H, Huang J, Mansson LE, Sham HP, McNagny KM, Vallance BA. Interleukin-11 reduces TLR4-induced colitis in TLR2-deficient mice and restores intestinal STAT3 signaling. Gastroenterology 2010;139:1277-88.

8. Bolstad BM, Collin F, Bretschneider J, Simpson K, Cope L. Bioinformatics and Computational Biology Solutions using R and Bioconductor. In: Gentleman R, Carey VJ, Huber W, Irizarry RA, Dudoit S, eds: Springer, 2005:33-48.

9. Irizarry RA, Hobbs B, Collin F, Beazer-Barclay YD, Antonellis KJ, Scherf U, Speed TP. Exploration, normalization, and summaries of high density oligonucleotide array probe level data. Biostatistics 2003;4:249--264.

10. Gentleman RC, Carey VJ, Bates DM, Bolstad B, Dettling M, Dudoit S, Ellis B, Gautier L, Ge Y, Gentry J, Hornik K, Hothorn T, Huber W, Iacus S, Irizarry R, Leisch F, Li C, Maechler M, Rossini AJ, Sawitzki G, Smith C, Smyth G, Tierney L, Yang JYH, Zhang J. Bioconductor: open software development for computational biology and bioinformatics. Genome Biol 2004;5:R80.

11. Goeman JJ, van de Geer SA, de Kort F, van Houwelingen HC. A global test for groups of genes: testing association with a clinical outcome. Bioinformatics 2004;20:93--99.

12. Pospisilik JA, Knauf C, Joza N, Benit P, Orthofer M, Cani PD, Ebersberger I, Nakashima T, Sarao R, Neely G, Esterbauer H, Kozlov A, Kahn CR, Kroemer G, Rustin P, Burcelin R, Penninger JM. Targeted deletion of AIF decreases mitochondrial oxidative phosphorylation and protects from obesity and diabetes. Cell 2007;131:476-91.
