# Supplementary material for: GUCY2C Opposes Systemic Genotoxic Tumorigenesis by Regulating AKT-Dependent Intestinal Barrier Integrity
Source: PLoS One. 2012 Feb 22;7(2):e31686. doi: 10.1371/journal.pone.0031686 (PMC3284579; doi:10.1371/journal.pone.0031686)
Supplement: Data S1 — Result of KEGG pathway 04530 (Tight Junction) between C57Bl6 Gucy2c+/+ and Gucy2c−/− mice. (HTML) [file pone.0031686.s004.html]

Division of Biostatistics, Thomas Jefferson University

|  |  |
| --- | --- |
| **Client:** Waldman Lab (PI: Scott Waldman, MD, PhD) | **Statistician:** Abhijit Dasgupta, PhD |

Back to index

## KEGG pathway 04530

**Description:**  Tight junction

**Number of probesets:**  91

**Number of genes:**  91

**Number of probesets (nominally) significant at 5%:** 29

| | name | Description | Influence | Expected | SD | z.score | colouring | | --- | --- | --- | --- | --- | --- | --- |  Cldn2 | claudin 2 | 1.3e+02 | 86.38 | 122.2 | 0.378 | high in KO | |
 Pard6b | par-6 (partitioning defective 6) homolog beta (C. elegans) | 1.2e+02 | 49.73 | 70.3 | 0.928 | high in KO | Actb | actin, beta, cytoplasmic | 1.0e+02 | 20.58 | 29.1 | 2.887 | high in WT | Ppp2r3a | protein phosphatase 2 (formerly 2A), regulatory subunit B'', alpha | 8.1e+01 | 13.76 | 19.5 | 3.429 | high in KO | Prkcd | protein kinase C, delta | 7.8e+01 | 51.24 | 72.5 | 0.362 | high in KO | Gnai3 | guanine nucleotide binding protein, alpha inhibiting 3 | 6.4e+01 | 12.14 | 17.2 | 3.019 | high in WT | Yes1 | Yamaguchi sarcoma viral (v-yes) oncogene homolog 1 | 6.3e+01 | 11.34 | 16.0 | 3.222 | high in WT | Cldn4 | claudin 4 | 6.2e+01 | 16.23 | 23.0 | 2.011 | high in KO | Csnk2a2 | casein kinase 2, alpha prime polypeptide | 6.1e+01 | 10.67 | 15.1 | 3.332 | high in KO | B230120H23Rik | RIKEN cDNA B230120H23 gene | 5.6e+01 | 12.53 | 17.7 | 2.457 | high in KO | Prkce | protein kinase C, epsilon | 5.5e+01 | 22.77 | 32.2 | 1 | high in KO | Prkcb1 | protein kinase C, beta 1 | 5.3e+01 | 9.68 | 13.7 | 3.155 | high in KO | F11r | F11 receptor | 5.2e+01 | 17.57 | 24.8 | 1.395 | high in KO | Myh14 | myosin, heavy polypeptide 14 | 5.0e+01 | 25.13 | 35.5 | 0.707 | high in KO | Tjp3 | tight junction protein 3 | 4.4e+01 | 19.33 | 27.3 | 0.915 | high in KO | Akt1 | thymoma viral proto-oncogene 1 | 4.3e+01 | 9.62 | 13.6 | 2.438 | high in KO | Ctnna1 | catenin (cadherin associated protein), alpha 1 | 4.2e+01 | 13.65 | 19.3 | 1.449 | high in KO | Nras | neuroblastoma ras oncogene | 4.2e+01 | 8.02 | 11.3 | 2.955 | high in KO | Cldn3 | claudin 3 | 4.1e+01 | 11.85 | 16.8 | 1.748 | high in KO | Myh9 | myosin, heavy polypeptide 9, non-muscle | 3.7e+01 | 6.91 | 9.8 | 3.051 | high in KO | Epb4.1 | erythrocyte protein band 4.1 | 3.6e+01 | 8.02 | 11.3 | 2.454 | high in KO | Prkch | protein kinase C, eta | 3.5e+01 | 5.96 | 8.4 | 3.502 | high in KO | Rhoa | ras homolog gene family, member A | 3.5e+01 | 34.58 | 48.9 | 0.007 | high in KO | Ppp2cb | protein phosphatase 2 (formerly 2A), catalytic subunit, beta isoform | 3.4e+01 | 6.68 | 9.4 | 2.937 | high in WT | Ppp2r1a | protein phosphatase 2 (formerly 2A), regulatory subunit A (PR 65), alpha isoform | 3.4e+01 | 5.69 | 8.0 | 3.511 | high in KO | Cttn | cortactin | 3.4e+01 | 8.82 | 12.5 | 1.995 | high in KO | Prkcc | protein kinase C, gamma | 3.3e+01 | 5.55 | 7.9 | 3.541 | high in WT | Csda | cold shock domain protein A | 3.2e+01 | 9.20 | 13.0 | 1.767 | high in KO | Actn4 | actinin alpha 4 | 3.1e+01 | 8.46 | 12.0 | 1.844 | high in KO | Tjp2 | tight junction protein 2 | 2.9e+01 | 10.92 | 15.4 | 1.19 | high in KO | Pard3 | par-3 (partitioning defective 3) homolog (C. elegans) | 2.7e+01 | 24.74 | 35.0 | 0.054 | high in KO | Pten | phosphatase and tensin homolog | 2.6e+01 | 6.29 | 8.9 | 2.236 | high in WT | Ppp2r1b | protein phosphatase 2 (formerly 2A), regulatory subunit A (PR 65), beta isoform | 2.6e+01 | 6.32 | 8.9 | 2.189 | high in KO | Cldn23 | claudin 23 | 2.6e+01 | 15.80 | 22.3 | 0.449 | high in KO | Llgl1 | lethal giant larvae homolog 1 (Drosophila) | 2.5e+01 | 5.87 | 8.3 | 2.296 | high in KO | Gnai2 | guanine nucleotide binding protein, alpha inhibiting 2 | 2.5e+01 | 6.89 | 9.7 | 1.844 | high in KO | Csnk2a1 | casein kinase 2, alpha 1 polypeptide | 2.5e+01 | 4.68 | 6.6 | 3.028 | high in WT | Inadl | InaD-like (Drosophila) | 2.3e+01 | 7.46 | 10.5 | 1.481 | high in KO | Jam2 | junction adhesion molecule 2 | 2.3e+01 | 13.63 | 19.3 | 0.484 | high in WT | Spnb2 | spectrin beta 2 | 2.2e+01 | 6.14 | 8.7 | 1.847 | high in KO | Cdk4 | cyclin-dependent kinase 4 | 2.2e+01 | 31.08 | 43.9 | -0.217 | high in KO | Ash1l | ash1 (absent, small, or homeotic)-like (Drosophila) | 2.1e+01 | 5.29 | 7.5 | 2.164 | high in KO | Kras | v-Ki-ras2 Kirsten rat sarcoma viral oncogene homolog | 1.8e+01 | 4.83 | 6.8 | 1.994 | high in KO | Cldn7 | claudin 7 | 1.7e+01 | 5.64 | 8.0 | 1.44 | high in KO | Epb4.1l1 | erythrocyte protein band 4.1-like 1 | 1.7e+01 | 5.28 | 7.5 | 1.52 | high in KO | Rras2 | related RAS viral (r-ras) oncogene homolog 2 | 1.5e+01 | 7.60 | 10.7 | 0.72 | high in WT | Sympk | symplekin | 1.5e+01 | 3.25 | 4.6 | 2.58 | high in KO | Prkcz | protein kinase C, zeta | 1.4e+01 | 12.82 | 18.1 | 0.079 | high in KO | Jam3 | junction adhesion molecule 3 | 1.3e+01 | 5.66 | 8.0 | 0.977 | high in WT | Magi1 | membrane associated guanylate kinase, WW and PDZ domain containing 1 | 1.3e+01 | 3.32 | 4.7 | 2.153 | high in KO | Hcls1 | hematopoietic cell specific Lyn substrate 1 | 1.1e+01 | 2.44 | 3.4 | 2.475 | high in KO | Akt3 | thymoma viral proto-oncogene 3 | 1.1e+01 | 6.52 | 9.2 | 0.479 | high in WT | Myh10 | myosin, heavy polypeptide 10, non-muscle | 1.1e+01 | 8.50 | 12.0 | 0.197 | high in KO | Cldn15 | claudin 15 | 1.1e+01 | 3.22 | 4.5 | 1.606 | high in KO | Rab13 | RAB13, member RAS oncogene family | 8.6e+00 | 3.76 | 5.3 | 0.916 | high in WT | Cldn1 | claudin 1 | 8.0e+00 | 2.14 | 3.0 | 1.954 | high in WT | Exoc3 | exocyst complex component 3 | 8.0e+00 | 5.59 | 7.9 | 0.309 | high in KO | Prkca | protein kinase C, alpha | 8.0e+00 | 2.20 | 3.1 | 1.868 | high in KO | Mpp5 | membrane protein, palmitoylated 5 (MAGUK p55 subfamily member 5) | 7.3e+00 | 3.55 | 5.0 | 0.754 | high in WT | Ctnnb1 | catenin (cadherin associated protein), beta 1 | 7.3e+00 | 1.55 | 2.2 | 2.638 | high in KO | Epb4.1l2 | erythrocyte protein band 4.1-like 2 | 7.0e+00 | 2.26 | 3.2 | 1.47 | high in KO | Ppp2r2c | protein phosphatase 2 (formerly 2A), regulatory subunit B (PR 52), gamma isoform | 6.9e+00 | 9.20 | 13.0 | -0.178 | high in WT | Myl6 | myosin, light polypeptide 6, alkali, smooth muscle and non-muscle | 6.3e+00 | 2.63 | 3.7 | 0.997 | high in WT | Ppp2r4 | protein phosphatase 2A, regulatory subunit B (PR 53) | 6.0e+00 | 2.22 | 3.1 | 1.219 | high in WT | Vapa | vesicle-associated membrane protein, associated protein A | 5.9e+00 | 2.72 | 3.8 | 0.836 | high in WT | Crb3 | crumbs homolog 3 (Drosophila) | 5.1e+00 | 1.25 | 1.8 | 2.173 | high in KO | Gnai1 | guanine nucleotide binding protein, alpha inhibiting 1 | 4.6e+00 | 4.01 | 5.7 | 0.099 | high in WT | Tjap1 | tight junction associated protein 1 | 4.3e+00 | 18.35 | 26.0 | -0.542 | high in KO | Epb4.1l3 | erythrocyte protein band 4.1-like 3 | 4.3e+00 | 1.17 | 1.7 | 1.86 | high in KO | Exoc4 | exocyst complex component 4 | 4.1e+00 | 1.49 | 2.1 | 1.228 | high in KO | Llgl2 | lethal giant larvae homolog 2 (Drosophila) | 3.7e+00 | 3.32 | 4.7 | 0.071 | high in KO | Cdc42 | cell division cycle 42 homolog (S. cerevisiae) | 3.5e+00 | 0.70 | 1.0 | 2.823 | high in WT | Actg1 | actin, gamma, cytoplasmic 1 | 3.5e+00 | 1.42 | 2.0 | 1.028 | high in KO | Csnk2b | casein kinase 2, beta polypeptide | 3.2e+00 | 1.28 | 1.8 | 1.062 | high in KO | Ppm1j | protein phosphatase 1J | 3.1e+00 | 20.01 | 28.3 | -0.596 | high in WT | Prkci | protein kinase C, iota | 3.1e+00 | 1.39 | 2.0 | 0.858 | high in KO | Ppp2r2d | protein phosphatase 2, regulatory subunit B, delta isoform | 3.0e+00 | 1.77 | 2.5 | 0.48 | high in WT | Src | Rous sarcoma oncogene | 3.0e+00 | 11.98 | 16.9 | -0.532 | high in WT | Tjp1 | tight junction protein 1 | 2.4e+00 | 0.82 | 1.2 | 1.414 | high in KO | Cask | calcium/calmodulin-dependent serine protein kinase (MAGUK family) | 1.9e+00 | 1.06 | 1.5 | 0.567 | high in WT | Akt2 | thymoma viral proto-oncogene 2 | 1.7e+00 | 1.39 | 2.0 | 0.166 | high in KO | Magi3 | membrane associated guanylate kinase, WW and PDZ domain containing 3 | 1.5e+00 | 1.25 | 1.8 | 0.152 | high in KO | Jam4 | junction adhesion molecule 4 | 9.9e-01 | 3.12 | 4.4 | -0.482 | high in WT | Ppp2ca | protein phosphatase 2 (formerly 2A), catalytic subunit, alpha isoform | 9.4e-01 | 0.28 | 0.4 | 1.629 | high in KO | 4930412D23Rik | RIKEN cDNA 4930412D23 gene | 7.4e-01 | 1.97 | 2.8 | -0.443 | high in WT | Hras1 | Harvey rat sarcoma virus oncogene 1 | 5.9e-01 | 1.73 | 2.5 | -0.467 | high in KO | Rras | Harvey rat sarcoma oncogene, subgroup R | 4.9e-01 | 4.09 | 5.8 | -0.622 | high in WT | Pard6a | par-6 (partitioning defective 6,) homolog alpha (C. elegans) | 4.5e-01 | 1.78 | 2.5 | -0.528 | high in KO | Spna2 | spectrin alpha 2 | 3.1e-01 | 1.22 | 1.7 | -0.528 | high in KO | Ocln | occludin | 1.4e-02 | 5.48 | 7.7 | -0.705 | high in WT | Pard6g | par-6 partitioning defective 6 homolog gamma (C. elegans) | 7.2e-03 | 1.39 | 2.0 | -0.703 | high in KO |

  

Back to index

/n
